# Microwave Pretreatment for the Extraction of Anthocyanins from Saffron Flowers: Assessment of Product Quality

**DOI:** 10.3390/antiox10071054

**Published:** 2021-06-29

**Authors:** Ana Álvarez, Sara Terreros, María J. Cocero, Rafael B. Mato

**Affiliations:** BioEcoUva, Research Institute on Bioeconomy, High Pressure Processes Group, Department of Chemical Engineering and Environmental Technology, University of Valladolid, 47011 Valladolid, Spain; ana.alvarez.martin.90@gmail.com (A.Á.); terreros94@gmail.com (S.T.); mjcocero@iq.uva.es (M.J.C.)

**Keywords:** saffron flowers, microwave pretreatment, solid–liquid ratio, kinetic analysis, color

## Abstract

The potential of saffron flowers as a source of polyphenols, and in particular anthocyanins, for the extraction of bioactive compounds and the production of a cyanic colorant was analyzed. A microwave pretreatment, prior to the conventional solid–liquid extraction process, was proposed as a feasible intensification step. The effectiveness of microwave pretreatment was assessed in terms of increased yield and improved quality of the final product. The operational variables studied were the pretreatment temperature (60–120 °C) and the solid–liquid ratio (0.30–0.50 g/mL). It was found that the addition of the microwave pretreatment to the conventional process allowed one to reduce extraction time by up to 12 times and to greatly improve the characteristics of the final product, using microwave energy densities as low as 0.16–0.54 kJ/mL. The extract quality was evaluated in terms of polyphenol richness (25% increase), product composition (80% of the anthocyanins was delphinidin), antioxidant capacity (boosted by the pretreatment) and color (variations in red and blue hue depending on conditions). To conclude, a microwave pretreatment in which the material is heated to a temperature of 65 °C with a solvent ratio of 0.30 g/mL was selected as the optimum to maximize process efficiency and product quality.

## 1. Introduction

Extracts from natural products have drawn the attention of food industry as an alternative to synthetic complements due to the antioxidant properties and health benefits of their main components: polyphenols [1,2]. Saffron biofloral residues have recently been nominated as an outstanding source of polyphenols, especially anthocyanins, due to their great potential and availability [3,4]. The production of the spice entails a large generation of residues. In particular, 68 L of flowers is needed to produce 1 kg of spice [5]. The petals and stigmas constitute this byproduct, which has no real use but can be valorized to improve the sustainability of the process.

A rich polyphenolic extract stemming from saffron flowers would be highly valued in the food industry as a natural colorant. Actual market trends show a predilection for such tints instead of synthetic ones because of their health benefits [6,7,8]. In the case of saffron flowers, it is not only their high content of antioxidant compounds that makes them a convenient food ingredient [9], but also their composition in minerals, dietary fiber, sugars, anions and organic acids [10,11]. In addition, anthocyanins are the phytochemicals that play the most important role as food additives due to their ability to scavenge superoxide radicals [11] and the cyanic hues they exhibit [12]. Blue colorants are rare in nature [7]. Few examples have been described in literature, such as the blue pigments isolated from Port red wine [13]. Most natural blue colorants are now under research [14]. Among the anthocyanins, delphinidin and petunidin derivatives have been reported to provide the cyanic colors (purple, lilac, mauve and blue) in saffron flowers. Malvidin is also present, but in a lower percentage [15,16,17].

According to previous work, there is a correlation between the concentration of active compounds and color [18], and thus color must be an important attribute to take into account. To characterize colors for industrial applications, the use of the CIELAB space is recognized as an international standard [18]. In this standard, a sphere is defined with axes that correspond to lightness (L*), green vs. redness (a*) and yellow vs. bluish (b*) [4]. Table 1 specifies the range of values that these variables can have and their meaning.

Most of the published works on the valorization of saffron flower residues focus on their in vivo properties, thus demonstrating the potential of the extracts as chemopreventive agents [8,19,20,21,22,23]. As for the extraction process, conventional liquid extraction is not very convenient, as it requires low solid–liquid ratios and long extraction times. Low solid–liquid ratios imply high solvent consumption, which is contrary to the principles of green chemistry [24]. Long extraction times (prolonged for more than one hour) seem quite excessive for the extraction of polyphenols from petals, since their structure is not that complex to represent a large mass transfer resistance. These two unfavorable characteristics favored the application of intensification techniques: high-pressure extraction (subcritical water and supercritical carbon dioxide) [3,25,26], enzymatic extraction [27], ultrasound-assisted extraction (UAE) [28] and microwave-assisted extraction (MAE) [28,29]. The latter is particularly interesting because of the good results published in the literature, but the extraction conditions have not been analyzed as extensively, and the few available results present a notable disparity. Table 2 presents a review of optimized extraction conditions for conventional solid–liquid extraction processes.

The different authors who have optimized the conventional extraction process have reported very different extraction conditions. While in some works the maximum yield is obtained with water as solvent and at ambient temperature [17], others find that a mixture of organic and water solvents at high temperature provides the best efficiency [11,30]. However, when the same temperature and ethanol percentage suggested as optimal for conventional solid–liquid extraction by Ahmadian-Kouchaksaraie et al. [30] were used by Da Porto and Natolino [28], the obtained yields using the same conventional extraction process were four-fold higher. On the other hand, all authors agree that an acidified solvent improves the extraction and stability of the active compounds, especially anthocyanins [33].

To clarify the effect of the microwaves on the extraction of bioactive compounds from saffron flowers, a new study is required where all the parameters of interest (polyphenol richness, product composition, antioxidant capacity and color) are analyzed and where not only the external operational variables are characterized but also the efficiency of the microwave heating. For this purpose, the absorbed energy density (Q_absorbed_, kJ/mL) [34] is reported in the present paper for all experiments. The product of the power and irradiation time selected for each experiment in the microwave oven determines the total amount of energy irradiated, but of this, only a fraction is absorbed by the sample. This fraction (absorption efficiency) usually ranges between 40% and 95%, depending on the experimental conditions (characteristics and geometry of the oven, amount and location of the sample in the oven, solvent composition, etc.). The calculation of the microwave absorbed energy, as described in Equations (1)–(4), helps elucidate whether the differences in the results reported by different authors may come from different qualities of raw materials or from different microwave absorption efficiencies, as the latter depends on many variables.

This work focused on the optimization of microwave extraction. However, in contrast to usual MAE processes, in this study, radiation has been used as a previous step to a conventional solid–liquid extraction. Bench-scale MAE experiments typically employ temperature control involving an initial power spike, to rapidly reach set temperature conditions, and an intermittent short power radiation to maintain those conditions [35]. This, together with the difficulties in adequately scaling a large cavity, has led to a preference for the use of microwaves as a brief pretreatment. By providing the extraction sample with a peak of microwave energy, a high temperature in a short time is achieved. Thus, the possible disruption of the matrix and the rapid extraction of the compounds take place [36]. In addition, due to their short duration, the degradation of thermosensitive compounds is very low if pretreatment is followed by rapid cooling [37]. Regarding the scale-up for the industrial application, the use of a low residence time in the microwave allows the design of a compact oven that can be easily implemented at a larger scale. In addition, previous work has shown that a microwave pretreatment improves not only the process yield, but also the polyphenol richness of the final product [38].

Thus, this work is aimed to develop an efficient and feasible scale-up process to obtain a functional antioxidant colorant.

## 2. Materials and Methods

### 2.1. Raw Material

Saffron flowers were donated by a local farmer. Saffron was collected before sunlight in October 2017 and manually processed. Floral byproducts were immediately frozen and stored at −20 °C. Before being used, flowers were thawed for one hour at 4 °C. Table 3 gathers the characterization of the raw material employed in this work. Moisture was determined gravimetrically by drying the sample until constant weight at 105 °C. Fat and extractives were quantified by a Soxhlet extraction with n-hexane, and ethanol and water, respectively. The protein content was computed by the Kjeldahl method, using a conversion factor of 6.25 [39]. Ashes were measured by means of the char formed at 550 °C. The fiber content is assumed to be 173.61 mg/g_SF_, which is the unanalyzed remainder, and is in accordance with literature data [40].

Saffron heat capacity was measured in a Setaram Micro DSC II microcalorimeter [41] from 20 to 85 °C and was found to be 3.89 ± 0.02 J/g K.

### 2.2. Chemical Reagents

APPH (2,2-azobis 2-amidopropane dihydrochloride), gallic acid, sodium fluorescein and trolox were purchased from Sigma. Acetonitrile, ammonium phosphate monobasic, chlorohydric acid, ethanol, Folin-Ciocalteu reagent, phosphate salts (NaH2PO4·2H2O and Na2HPO4·12H2O), phosphoric acid, potassium chloride, sodium acetate and sodium carbonate were obtained from Panreac. HPLC standards catechin, delphinidin, epicatechin, gallic acid, malvidin and quercetin were bought from Extrasynthese, except for kaempferol which was purchased from TCI. A Millipore unit was used to purify the water used as solvent.

### 2.3. Extraction Procedure

A conventional solid–liquid extraction process was used as a control reference to assess the influence of the microwave pretreatment. In all experiments, an amount of 30 g of saffron flowers was weighed and mixed with the corresponding volume of solvent to achieve the desired solid–liquid ratio. An ethanol–water mixture was used as solvent. Ethanol was chosen as the organic solvent because of its GRAS (generally recognized as safe by the FDA) certification [5]. To improve the yield and stability of the anthocyanins, water acidified to pH = 2 with hydrochloric acid was used [11].

Once the saffron flowers were mixed with the solvent, they were homogenized for 5 min at room temperature and stirred gently. The extraction itself was set to begin when the flask was place in a thermostatic bath at the set temperature and with vigorous stirring.

For experiments in which a microwave pretreatment was added to the process, it was integrated after the homogenization and before the thermostatic and stirring bath. The microwave pretreatment was carried out in a CEM Discover One unit (CEM Corp). It was devised as an intense and short pretreatment, ease to scale up for a future industrial application. Thus, the maximum power, 300 W, was irradiated for shorter times than traditional MAE, between 40 and 145 s, to reach temperatures of 60, 80, 100 and 120 °C. These temperatures were measured with an optical fiber (FOTEMP4, OPTcon GmbH), weekly calibrated at 0 °C with a bath of ice and distilled water. The pretreatments at 60 and 80 °C were conducted in an open vessel round bottom flask, whereas for the 100 and 120 °C, a pressure cell was needed to avoid sample evaporation by boiling. A 100 mL QianCap glass pressure vessel (QLabtech) was used.

Unlike most published work on MAE, microwave pretreatment has been quantified in terms of absorbed energy density rather than using the radiation conditions selected in the oven. The absorbed energy density (Q_absorbed_) was calculated by means of an energy balance, based on the two main contributions: sensible heat and latent heats, as expressed in Equation (1). Losses to the environment have been dismissed in accordance to Sólyom’s conclusions [34].

(1) $$Q_{\mathrm{absorbed}}=Q_{\mathrm{sensible}}+Q_{\mathrm{latent}}$$

The sensible heat (Q_sensible_) was evaluated using Equation (2), which calculates the energy required to increase the temperature of the extraction media (solvent and saffron flower) to the final value specified in the pretreatment.

(2) $$Q_{\mathrm{sensible}}=\sum m\;C_{p}\;\Delta T$$

where m is the mass of every component in the sample, C_p_ its specific heat, and ΔT the temperature rise experienced during the pretreatment.

The latent heat (Q_latent_) is calculated according to Equation (3).

(3) $$Q_{\mathrm{latent}}=n_{\mathrm{evaporated}}\;\lambda$$

where λ is the solvent vaporization heat.

The number of moles of solvent evaporated (n_evaporated_) was calculated using two different procedures, depending on whether the pretreatment was carried out in an open vessel or in a pressure cell. For the open vessel experiments (pretreatments up to 60 and 80 °C), it was calculated by weight loss. However, when the specified pretreatment temperature exceeded the normal boiling point of the solvent (pretreatments up to 100 and 120 °C), pretreatment had to be carried out in a closed vessel. In this case, the evaporated solvent accumulates in the closed gas space, increasing the pressure inside the vessel. The number of evaporated moles in this case was calculated from the registered maximum final pressure (P) reached during the pretreatment, assuming ideal gas behavior:

(4) $$n_{\mathrm{evaporated}}=\frac{PV_{0}-RTn_{0}}{RT-\frac{P}{\rho_{\mathrm{mol}}}}$$

where V_0_ is the empty cell volume, R the gas constant, n_0_ the number of air moles in the initial gas space, T the maximum final temperature, V_0_ the volume of the gas space (cell minus liquid volume), and ρ_mol_ the molar density of the liquid solvent. Liquid composition changes due to evaporation were neglected since only a very low fraction of the solvent is evaporated (between the 0.18% and 0.35%).

Once the pretreatment was finished, the media was rapidly cooled down using an ice-water bath. In those cases where the solid–liquid ratio used in the pretreatment was different from the one used in the subsequent extraction stage, the extra cold solvent was added just after the pretreatment to accelerate the cooling stage. Previous work has shown that the use of different solid–liquid ratios can improve extraction efficiency [38]. The medium was then placed in the thermostatic bath, and the process continued as in a conventional solid–liquid extraction for 180 min. Liquid samples were taken throughout the process to determine the extraction kinetics and richness. The calculated concentration data (c_cal_) were adjusted to the experimental values (c_exp_) using the first order kinetic equation described in Equation (5), minimizing the average relative deviation (ARD) defined in Equation (6).

(5) $$c_{\mathrm{cal}}=c_{o}+c_{f}\left[1-\exp\left(-\mathrm{kt}\right)\right]$$

(6) $$\mathrm{ARD}=\frac{1}{n}\sum \left|\frac{c_{\exp}-c_{\mathrm{cal}}}{c_{\exp}}\right|$$

Concentrations are expressed in mg/g_dry saffron flowers_. The regressed parameters in Equation (5) were the initial concentration c_0_, the pre-exponential factor c_f_, and the rate constant k.

The use of this kinetic model makes it easy to assess the extraction efficiency of a particular extraction process by means of the derived parameters initial extraction rate (u_0_) and yield (c_∞_). The initial extraction rate, calculated by Equation (7), represents the extraction rate at t = 0 (start of the extraction period after the pretreatment). The higher this parameter is, the shorter the time required to reach maximum yield.

(7) $$u_{o}={\frac{\partial c_{\mathrm{cal}}}{\partial t}|}_{t=0}=c_{f}k$$

The yield (c_∞_, Equation (8)) determines the maximum concentration of polyphenols that can be achieved after endless extraction.

(8) $$c_{\infty }=\underset{t\to \infty }{\mathrm{Lim}}c_{\mathrm{cal}}=c_{0}+c_{f}$$

The polyphenol and anthocyanin richness were also calculated to characterize the extract. These parameters indicate the fraction of polyphenols or anthocyanins in the total amount of solid extract product, since they are also mixed with sugars, fibers, etc. Since extracts are usually commercialized as solid products, their specific richness is crucial as it determines the quality and price of the final product. Richness was expressed in mg/g_Dry Extract_, and it was calculated from the polyphenols or anthocyanins concentration (mg/g_Extract_) and the solid extract residue (g_Dry Extract_/g_Extract_).

### 2.4. pH Measurement

pH throughout the extraction was recorded by a Jenway 3505 pH meter.

### 2.5. Extract Characterization

#### 2.5.1. Total Polyphenol Content

The Folin–Ciocalteu method was used to determine the total polyphenol content (TPC). In brief, a volume of 40 µL of sample was mixed with 3000 µL of distillate water and 200 µL of Folin–Ciocalteu reagent. After 5 min, 600 µL of 20% sodium carbonate solution was added. The mixtures were left for 30 min at 40 °C before recording their absorbance at 765 nm (Shimadzu UV/VIS spectrophotometer). Results were expressed in gallic acid equivalents (mg_GAE_/g).

#### 2.5.2. Anthocyanin Content

The pH differential method was used to quantify the anthocyanin content (AC). Samples were diluted in two buffers: one at pH = 1 of potassium chloride 0.025 M and another at pH = 4.5 of sodium acetate 0.4 M. The increase in absorbance between 520 and 700 nm was used together with an extinction coefficient of 26,900 L/(mol cm) to calculate the anthocyanin content, expressed in cyaniding-glucoside equivalents (mg_CGE_/g).

#### 2.5.3. Solid Residue

The solid residue was determined gravimetrically by drying the extracts at 105 °C overnight.

#### 2.5.4. Antioxidant Capacity

Antioxidant activity (AAO) was computed as the ability of the extracts to quench oxygen radicals from the decomposition of AAPH (α,α′-azodiisobutyramidine dihydrochloride, 240 mM). Sodium fluorescein (100 nM) was used as probe and trolox as the standard (200 µM). Phosphate buffer (10 mM, pH = 7.4) was used as solvent in the assays.

In a 96-well plate, 150 µL of fluorescein and 25 µL of sample (standard or diluted extract) were poured. After 30 min incubation at 37 °C, 25 µL of APPH was added. Fluorescence was then recorded in a Fluorstar Optima (BMG Labtech) at an emission wavelength of 530 ± 25 nm and excitation wavelength 485 ± 20 nm, until the signal variation was null. Trolox calibration was repeated for each assay, and samples were measured at least six times to minimize experimental error. Results were expressed as trolox equivalents (µ_molTE_/g).

#### 2.5.5. HPLC

Catechin, delphinidin, epicatechin, gallic acid, kaempferol, malvidin and quercetin were quantified. A Waters e2695 separation module and a Waters 2998 photodiode array detector (DAD) were employed. A previous method was used as reference [42]. A volume of 20 µL of sample was injected into the 1 mL/min eluent flow and passed through a Teknokrima C18 (250 × 4.6 mm, 5 µm) column dotted with an OptiGuard precolumn at 35 °C. The eluents used were (A) ammonium phosphate monobasic 50 mM and pH = 2.6, (B) a mixture of 80% acetonitrile and 20% of eluent A, and (C) acidified water with phosphoric acid to pH = 1.5. The eluent gradient is detailed in Table 4. DAD signals were recorded at 360 and 520 nm as well as the UV/VIS spectra. As they were very complex samples, the whole spectrum was recorded to compare it with the standard. The gallic acid was measured at 280 nm. Compound identification was performed by comparing retention time and UV/VIS spectra with the standards.

### 2.6. Color Measurement

A spectrophotometric method [43] was used to determine extract colors. This method allowed to compute color coordinates in the XYZ space by applying Equations (9)–(11) with the measured transmittances (T) at the selected wavelengths (see Appendix A). These measurements correspond to an illuminant C and a 2° standard observer.

(9) $$X=0.03269\;\sum T_{X}$$

(10) $$X=0.03333\;\sum T_{Y}$$

(11) $$X=0.03938\;\sum T_{Z}$$

To avoid errors due to different dilutions of the extracts, the samples were dried and diluted again to have a similar concentration. Liquid samples were concentrated in a rotary evaporator (Heidolph) to remove the ethanol and freeze-dried at −50 °C and 0.1 bar (Telstar LyoQuest). The dried extracts were then rediluted to a concentration of about 500 ppm. This final solution was placed in a ShimadzuUV/vis spectrophotometer where a transmittance scan from 700 to 400 nm was performed. Since CIELAB is the most extended color space in food industry, XYZ coordinates were transformed into the CIELAB ones (L*, a*, b*) by means of Equations (12)–(14). In these expressions, X_n_, Y_n_ and Z_n_ represent the illuminant C tristimulus, which for the conditions used here have values of X_n_ = 98.0681, Y_n_ = 100 and Z_n_ = 118.2313.

(12) $$L*=116{\left(Y/Y_{n}\right)}^{1/3}-16$$

(13) $$a*=500\left[{\left(X/X_{n}\right)}^{1/3}-{\left(Y/Y_{n}\right)}^{1/3}\right]$$

(14) $$b*=200\left[{\left(Y/Y_{n}\right)}^{1/3}-{\left(Z/Z_{n}\right)}^{1/3}\right]$$

It must be noticed that, since no direct measurement of the final product was made (but a dilution), these measurements only allow for comparison of color changes. For ease of comparison, the total color difference (∆E in Equation (15)) was used. In this equation, the subindex i denotates the analyzed sample, while 0 stands for the control. To assess the influence of microwave pretreatment on product color, conventional solid–liquid extraction (without microwaves) was used as the control.

(15) $$\Delta E=\sqrt{{\left(L_{i}^{*}-L_{0}^{*}\right)}^{2}+{\left(a_{i}^{*}-a_{0}^{*}\right)}^{2}+{\left(b_{i}^{*}-b_{0}^{*}\right)}^{2}}$$

For an easy CIELAB parameters interpretation, the software JMP has been employed to estimate ellipses with a 90% coverage of the experimental dispersion.

### 2.7. Experimental Design and Statistical Analysis

The pretreatment variables studied were the solid–liquid ratio (S:L) and the final temperature at the end of the microwave pretreatment (T_MW_). Experimental conditions are gathered in Table 5. A factorial experimental design was employed. ANOVA tables with a confidence of 95% (*p*-value of 0.05) were developed to determine significant effects. To compare the influence of variables in a similar range, coded variables have been used (−1/0/+1). They are identified in parentheses after the variable values. Design Expert software has been used for this analysis.

## 3. Results and Discussion

### 3.1. Conventional Solid–Liquid Extraction

**Figure 1:**
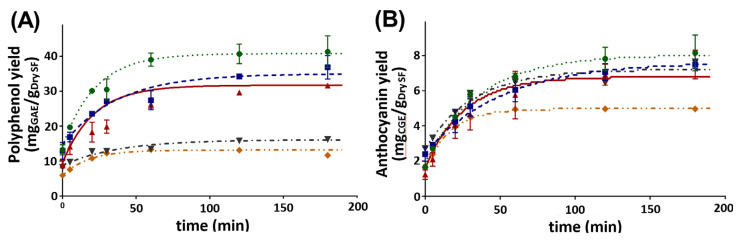
Solid–liquid ratio influence on conventional solid–liquid extraction for (**A**) polyphenol and (**B**) anthocyanin extraction. ● S:L = 0.10 g/mL, ■ S:L = 0.20 g/mL, ▲ S:L = 0.30 g/mL, ▼ S:L = 0.50 g/mL, ♦ S:L = 0.75 g/mL.

The experimental conditions (temperature, solvent composition and solid–liquid ratio) used to carry out the conventional solid–liquid extractions, both in the control experiments and to complete the extraction in the experiments with microwave pre-treatment, were selected based on the analysis of three inputs: (1) the optimal conditions reported in the literature and listed in Table 2 for conventional extraction processes; (2) industrial and scale-up aspects, such as solvent consumption, extract concentration or the use of saffron flowers without any conditioning (grinding or drying); and (3) some preliminary experimental work, presented below in Figure 1, to evaluate the influence of the solid–liquid ratio on extraction efficiency.

Regarding extraction temperature, previous authors (Table 2) suggested the convenience of employing extraction temperatures of 30 and 60 °C. Similar polyphenol yields and richness were achieved in both cases. However, an anthocyanin yield decrease was found at 60 °C due to the thermal degradation of these compounds after 40 min of extraction. For these reasons, 30 °C was selected as the extraction temperature.

As for the solvent, water was found to greatly enhance the extraction of anthocyanins but not polyphenols to the same extent. Therefore, the use of only water as a solvent was discarded in favor of a 50% ethanol:water solution (water acidified at pH = 2), since this mixture allowed one to achieve a convenient compromise between polyphenol and anthocyanin extraction yields.

The solid–liquid ratio was the most influential factor; therefore, a thorough analysis was performed. Ratios ranging from 0.10 to 0.75 g/mL were studied. Results can be found in Figure 1.

Different final yields were obtained for total polyphenols, decreasing with increasing S:L ratio. The influence of solvent composition on mass transfer is considered as the reason for this behavior. For low S:L values (large volume of solvent), depletion of the extractives could be achieved because of the large concentration gradient between the solid and the diluted solvent. However, these extracted polyphenols were also highly diluted in the solvent; therefore, a low concentrated liquid product was finally obtained. By contrast, when a low solvent volume was used (high S:L ratio), a smaller concentration gradient drove the extraction, and consequently, a smaller fraction of polyphenols was extracted. As the polyphenols accumulated in the low-volume liquid phase, a more concentrated extract could be obtained.

**Figure 2:**
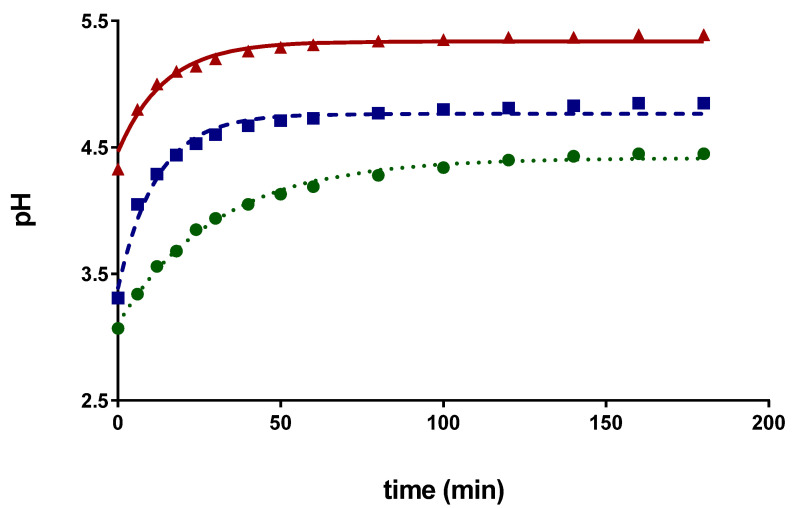
pH evolution during the extraction for a solid liquid ratio of ● S:L = 0.10 g/mL, ■ S:L = 0.20 g/mL, and ▲ S:L = 0.30 g/mL.

Another aspect considered was the likely influence of the acidified solvent. Since acidified water was employed as a solvent constituent, the presence of these ions could interfere with the structure of saffron, reducing the binding of polyphenols and enhancing their release. Figure 2 represents the pH evolution during the extraction at different S:L ratios. Lower ratios entailed a larger solvent consumption, and therefore, the pH maintained during the extraction closer to that of the fresh solvent (pH = 2). The greater concentration of the hydronium ion present in the extraction media improved the extraction of compounds by acid hydrolysis.

Unlike total polyphenols, anthocyanin extraction achieved almost the same final yield regardless of the solvent (except in the case of 0.75 g/mL, which was proved to be limited by solubility). This indicated that anthocyanin was easily released from saffron flowers.

Finally, a S:L ratio of 0.30 g/mL was selected as the optimum. Although this ratio did not lead to the highest yield of polyphenols (23% less was achieved), it made it possible to obtain a much more concentrated extract. Specifically, 230% more polyphenols was obtained per liter of solvent (9.6 mgGAE/mL compared to 4.1 mgGAE/mL when 0.10 g/mL was used) and 276% more in the case of anthocyanins (2.2 mgGGE/mL compared to 0.8 mgGGE/mL when 0.10 g/mL was used). This increase is interesting for favoring the profitability and sustainability of the process, since much less energy is required to evaporate the solvent to obtain the final solid extract.

### 3.2. Microwave Pretreatment Extraction

This section analyzes the effect on the kinetics of adding microwave pretreatment to conventional solid–liquid extraction, the main results of which are shown in Table 5 and Table 6.

The table allows one to identify the effects of each variable (linear and quadratic), as well as possible interactions.

The operating variables of the pretreatment, S:L and T_MW_, determined the total microwave energy absorbed by the extraction media, shown in Table 5. This is the value that must be considered when designing an industrial scale oven. The absorbed energy density in these experiments ranged between 0.23 and 0.64 kJ/mL.

The conventional solid–liquid extraction (without a microwave pretreatment) was taken as the control reference to assess the efficiency of the pretreatment. The corresponding values are also included in Table 5 and Table 6.

#### 3.2.1. Initial Extraction Rate

**Figure 3:**
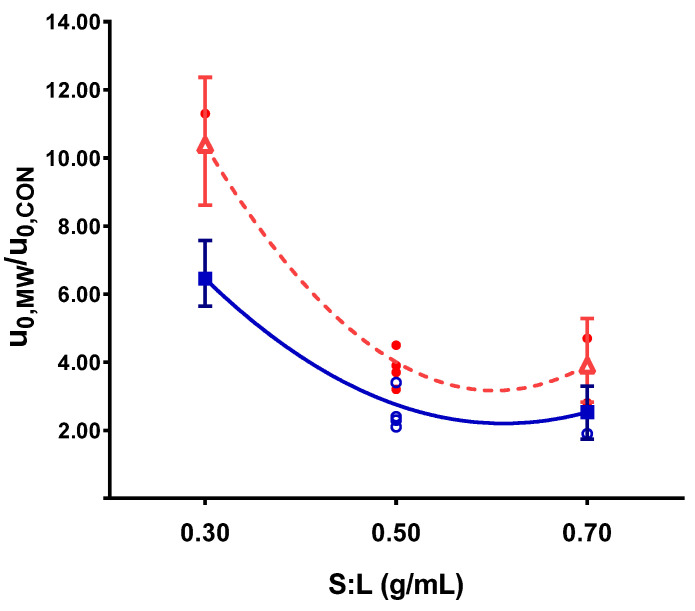
Solid–liquid ratio influence on initial extraction rate respect to the control for a pretreatment temperature of 80 °C (■ polyphenol extraction, **Δ** anthocyanin extraction, **○** and ● experimental points for polyphenols and anthocyanins, respectively).

**Figure 4:**
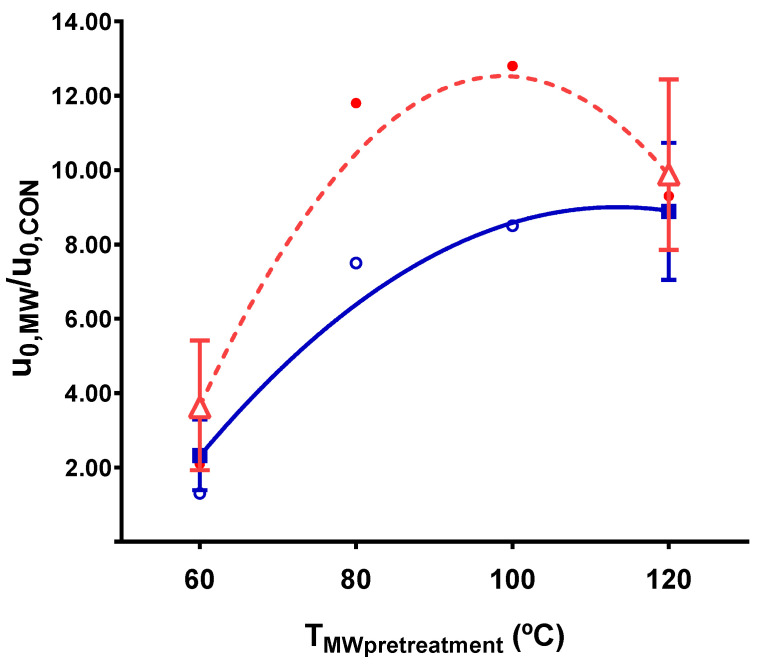
Pretreatment temperature influence on initial extraction rate respect to the control for a solid–liquid ratio of 0.30 g/mL (■ polyphenol extraction, **Δ** anthocyanin extraction, **○** and ● experimental points for polyphenols and anthocyanins, respectively).

The most relevant effect of the of microwave pretreatment application was the significant acceleration of the initial extraction rate, both in polyphenols and anthocyanins. The acceleration caused by pretreatment ranged from 2 to 12 times the conventional value. How the S:L ratio and the pretreatment temperature influenced this acceleration can be observed in Figure 3 and Figure 4, respectively.

**Figure 5:**
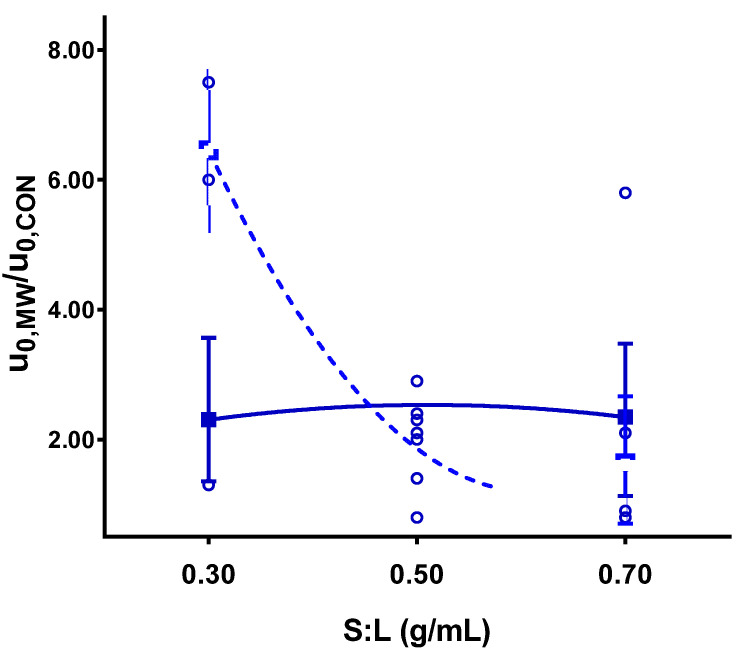
Coupled effect of S:L ratio and pretreatment temperature on the polyphenols initial extraction rate at 60 °C (■) and 120 °C (**□**).

Lower S:L ratios led to faster extractions. The same behavior, although less pronounced, was also observed in the conventional solid–liquid extraction (Figure 5). This effect was explained in terms of the concentration gradients. Lower S:L ratios implied larger solvent volumes and more diluted extracts, leading to a higher mass transfer driving force. Nevertheless, it was shown that the application of the pretreatment significantly increased the initial extraction rate (u_0_), demonstrating the enhancement of mass transfer due to microwave pretreatment.

As for the pretreatment temperature, the initial extraction rate accelerated with temperature up to 100 °C. At higher temperatures (120 °C), the polyphenol content showed no further improvement, while the anthocyanin content appeared to show a decrease, despite the experimental error. Considering the anthocyanin richness (results shown in Table 6), a 17% decrease in anthocyanin richness was observed at 120 °C, which corroborated the hypothesis of thermal degradation. Thus, it can be stated that as the pretreatment temperature increased up to 100 °C, the extraction of polyphenols and anthocyanins accelerated rapidly, compensating their thermal degradation rate. However, at higher pretreatment temperatures, the influence of degradation exerted a greater influence on the balance than the increase in extraction yield. This agrees with [10], who reported that 70–90 °C was the best temperature range to maintain the antioxidant properties of saffron flowers during a drying compared to higher temperatures (110 and 125 °C). On the other hand, it contrasts with the results of [37] on the stability of grape pomace anthocyanins, where 10% degradation was reported after 3 min at 120 °C. In this work, the extraction media was exposed to that temperature for 2.4 min. In the present work, almost twice the thermal degradation was observed for saffron anthocyanins with respect to the value observed by Solyom for grape anthocyanins, which may be due to differences in the raw material matrix.

It is worth considering the coupled effect of the interaction between the S:L ratio and the pretreatment temperature on the initial extraction rate. No difference was found in the initial extraction rate at low pretreatment temperatures (Figure 5), independently of the solid–liquid ratio. However, at higher temperatures, a substantial acceleration was observed for low S:L ratios. In this case, both variables contribute to improve the extraction process: the high temperature accelerated the rate, and the low S:L ratio ensured a large driving force that enhanced mass transfer. This interaction was significant for polyphenol extraction but was not as crucial for anthocyanins (a significance of 10%), although it showed the same tendency.

The application of microwave pretreatment significantly improved the initial extraction rate, allowing a considerable reduction of the extraction time.

#### 3.2.2. Extraction Yield

Extraction yield (polyphenols extracted from the saffron flowers) was not affected by the addition of the microwave pretreatment. The same yield than in the conventional solid–liquid extraction was obtained for both polyphenols (around 26.19 ± 2.26 mgGAE/gDry SF) and anthocyanins (around 6.92 ± 1.30 mgCGE/gDry SF). Thus, it can be concluded that saffron flowers were completely exhausted by the extraction.

#### 3.2.3. Product Richness

**Figure 6:**
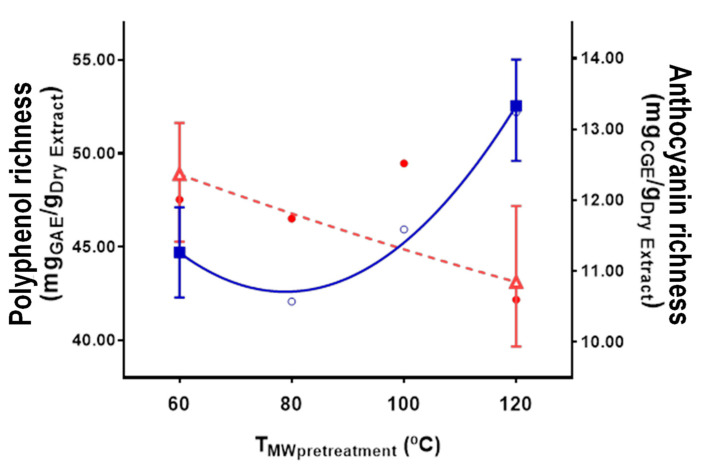
Polyphenol (■) and anthocyanin (**∆**) richness in the extraction with microwave pretreatment at 0.30 g/mL S:L ratio (○ polyphenol and ● anthocyanin experimental points).

Polyphenol and anthocyanin richness (the concentration of active compounds obtained in the dry product after solvent evaporation of the extract) remained constant throughout the extraction. This meant that the extraction of active compounds and undesired compounds (sugars, fibers, etc.) took place always in the same kinetics ratio. Pretreatment resulted in the same acceleration in both desired and undesired compounds, unlike in the case of grape pomace [38]. In this latter case, polyphenol extraction was substantially enhanced by pretreatment but no other compounds extraction, which decayed polyphenol richness. This allowed one to find an optimal time at which polyphenols were extracted but other compounds that decay polyphenol richness were not. However, in the case of saffron flowers, it was not possible to find an optimal time to improve polyphenol richness. To facilitate the analysis of the influence of the experimental conditions, the average richness of the product throughout the extraction was used. Pretreatment temperature was found to be significant according to the analysis of variance. However, an exhaustive analysis of this variable cannot be performed due to the large experimental error, especially for anthocyanins, as it can be seen in Figure 6. Nonetheless, from these results, it can be concluded that polyphenol richness improved with higher microwave pretreatment temperatures. Considering that the conventional extraction provides a richness of 41.82 mg_GAE_/g_Dry extract_, polyphenol richness was especially enhanced when temperatures above the solvent boiling point (combination of microwaves and pressure) were employed in the pretreatment. A 9.8% and a 24.8% rich improvement stood out at 100 °C and 120 °C, respectively. On the other hand, no improvement in anthocyanin richness was observed due to microwave pretreatment but a decrease of 8.8% caused by thermal degradation due to exposure to higher temperatures.

### 3.3. End Product Characterization

In addition to the richness of the product, there are other characteristics that determine the quality of the end product.

#### 3.3.1. Product Composition

As for the identification of individual polyphenols, the presence of gallic acid, catechin, epicatechin, quercetin, kaempferol and maldivin has been reported in similar extracts [44,45], but they have not been detected in this study. On the other hand, delphinidin was found in a very large concentration. It represented 80% of the total anthocyanins detected by HPLC at 520 nm wavelength [11] and 15% of the total polyphenols. Delphinidin concentrations obtained in microwave pretreatment varied from 5.03 to 7.89 mg/g_Dry Extract_. These concentrations represent an average improvement of 28% over the conventional extract. The extraction conditions used to maximize delphinidin richness agreed with the previous described tendencies to optimize anthocyanin extraction. In essence, it can be said that, although the richness of the extract can be considered low (an average of 5% for polyphenols and 1% for anthocyanins), the fact that a high-value compound such as delphinidin was present in such high concentrations greatly enhances the quality of the final product. The addition of a purification step would make it possible not only to obtain an extract rich in polyphenols but also a product specifically rich in delphinidin.

The characterization of the extract was concluded with an analysis of its antioxidant capacity. Most of the antioxidant activities of saffron flower extracts reported in other works are expressed as the extract ability to inhibit DPPH [21,30]. However, this kind of radical is scarce in biological systems [46]; therefore, the capacity to quench alkoxyl radicals was assessed instead. Again, improvements could be observed due to the addition of microwave pretreatment. An average improvement of 36% was found, although the antioxidant activity increased to more than 100% over conventional extraction without pretreatment when mild pretreatment conditions (low pretreatment temperature and low solid–liquid ratio) were used.

#### 3.3.2. Color

**Figure 7:**
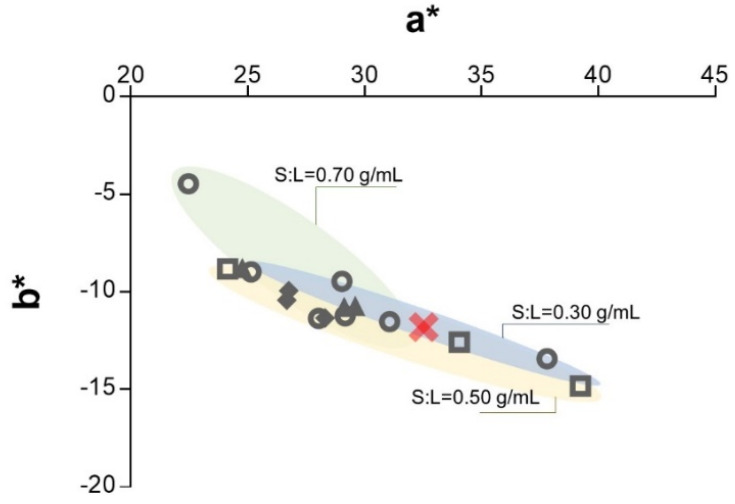
CIELAB parameters a* and b* as a function of the pretreatment temperature: **×** conventional solid–liquid extraction, □ TMW = 60 °C, ○ TMW = 80 °C, ▲ TMW = 100 °C, and ♦ TMW = 120 °C.

Because one of the likely applications of this extract is as food colorant, variations in color have been analyzed. The three CIELAB parameters considered for this purpose (L*, a* and b*) are gathered in Table 5. It must be noticed that color was measured in a solution prepared with the dried extract; therefore, these values can only be used to identify differences, not to characterize final extract color. However, since the dry extract is expected to be the preferable form of the commercial product, this may be considered the analysis of interest. From the results, it can be concluded that the application of microwave pretreatment only slightly influenced the color of the extract. An average total color difference of 5.96 was found when microwaves were employed compared to the conventional solid–liquid extraction. The main difference was found in chroma, while hue (L*) was almost constant. However, it is worth noting that each of the CIELAB parameters, L*, a* and b*, showed a relationship with anthocyanin content, as already observed by other authors [47]. Luminosity decreased with the concentration of anthocyanins (darker extracts were obtained), and red hue was improved (a* tended to higher values), as well as blue (b* tended to negative). According to the optimal anthocyanin extraction conditions, experiments performed with a low S:L ratio and low pretreatment temperature (0.30 g/mL and 60 °C, which entailed an absorbed energy density of 0.23 kJ/mL) gave a high anthocyanin richness and, consequently, the highest color increase in terms of red and blue. An intense violet extract was obtained. If more pronounced conditions were employed (80–100 °C and 0.30–0.50 g/mL, with absorbed energy density between 0.39 and 0.58 kJ/mL), a blue hue similar to that of the conventional extraction was found, but a lower a* parameter was obtained, indicating a tendency to green. These results are shown in Figure 7. The most severe conditions (120 °C and 0.50–0.70 g/mL, with absorbed energy densities between 0.58 and 0.64 kJ/mL) caused a deviation from the desired color. A brighter and more yellow-green colored extract was obtained compared to the one obtained in the conventional extraction. A tendency to yellow in saffron flower extracts was also observed by other authors when the extract was encapsulated [4] or when saffron flowers were dried [10]. The latter attributed the color change to thermal degradation of anthocyanins, which seems to be a reasonable justification. Therefore, if a bluer extract is sought, a mild pretreatment (according to optimal anthocyanin extraction conditions) should be used.

### 3.4. Extraction Optimization

Optimization was performed using response surface methodology with the statistical analysis software DesignExpert v. 11 (StatEase, Minneapolis, MN, USA). The operating variables S:L ratio and pretreatment temperature were optimized to maximize extraction kinetics and to improve the characteristics of the final product (richness and color). The optimization method allows for a multiple goal target. To do this, the different variables of interest to be optimized must be ranked according to the specific ‘Objective’ (maximize/minimize/none) and the weight of each variable in the final objective function (desirability function) must be quantified in terms of ‘Relative importance’ (0–5). Greater relative importance (value = 5) was attributed to richness and color (blue and red), since these factors are closely related to the final application of the product. Kinetic improvements (extraction rate rather than yield) were considered to be of medium importance (value = 4), and a low-medium importance (value = 3) was chosen for yields and antioxidant activities. Results are presented in Table 7 in the ‘Theoretical optimum’ column:

New extraction experiments were then carried out using this ‘Theoretical optimum’ conditions (S:L ratio = 0.30 and pretreatment temperature = 66 °C). The experimental results (‘Experimental optimum’ column in Table 7) corroborated the overall convenience of adding a microwave pretreatment prior to conventional extraction. Extraction efficiency and final product quality were boosted. The initial extraction rate (u_0_) was the most pronounced improvement. It was accelerated 12 and 16 times for polyphenols and anthocyanins extraction, respectively. An improvement in the end product was also achieved. The result was an extract 30% richer in polyphenols and, more importantly, 50% more concentrated in anthocyanins. Such enhancement in polyphenol concentration was also reflected in the CIELAB color parameters: a darker and bluer extract was obtained.

## 4. Conclusions

The use of a microwave pretreatment, consisting of a short but intense heating step followed by rapid cooling before conventional solid–liquid extraction, has been shown to accelerate the extraction of polyphenols while minimizing their degradation. The variables analyzed were the maximum temperature reached and the solid–liquid ratio used, both referring to pretreatment. The effect of pretreatment was compared with a conventional extraction (without microwave pretreatment), the conditions of which were also optimized in this work. In the optimization study of the conventional solid–liquid extraction, the ratio of saffron flowers to solvent (S:L ratio) proved to be of vital importance. It determined whether a concentrated liquid product was obtained (high S:L ratios) or whether the polyphenol content in the saffron flowers was completely depleted (low ratios). The acidity of the solvent used was also considered significant, as it could have contributed to the hydrolysis of the petal structure, thus facilitating the release of the active compounds. Finally, a ratio of 0.30 g/mL was selected for the conventional solid–liquid extraction, as it implied a balance between solvent consumption, polyphenol extraction and liquid-extract concentration. It also ensured maximum yield of anthocyanins, which were the compounds of greatest interest. This extraction medium, at a temperature of 30 °C, was also used for all extractions after microwave pretreatment.

The main advantages obtained with the application of microwave pretreatment were the reduction of the extraction time and the improvement of the quality of the final product. The initial extraction rate was the most affected parameter. It was greatly accelerated when moderately high temperatures and low solid–liquid ratios were used. This improvement made it possible to obtain a high yield in 5 min, instead of almost an hour, as required by the conventional process. Moreover, there was an increase in product quality in terms of richness, composition, antioxidant activity and color. It was found that the richness in polyphenols and anthocyanins (proportion of active compounds in the final dry product) showed opposite trends. The former increased with higher pretreatment temperatures, up to 25% at 100 °C, while anthocyanin richness decreased by 9% at the same temperature increase, due to thermal degradation. Thus, optimal anthocyanin extraction conditions were established at a low pretreatment temperature and at low S:L ratio. These conditions also maximized the antioxidant activity of the extract (130%) and the color of the final product. With respect to the latter, mild conditions enhanced the red and blue tones, while more severe conditions caused the extract to move away from the desired tannic hue to a greener, yellowish product, probably due to thermal degradation. Finally, as for the composition of the extract, delphinidin was the main compound detected. It represented 80% of total anthocyanins and 15% of total polyphenols. Such specificity raised the value of the extract, as it can be easily converted into a delphinidin extract by the addition of a purification step.

In summary, it was concluded that a gentle microwave pretreatment (with a ratio of 0.30 g/mL and irradiating the medium up to a temperature of 65 °C) was very convenient as it allowed for the obtaining of a high-quality product in an efficient extraction process.

## Figures and Tables

**Table 1:** CIELAB parameters meaning.

Lightness		Hue			
L = 0	black	a* > 0	red	b* > 0	yellow
L = 100	white	a* < 0	green	b* < 0	blue

**Table 2:** Yields and optimum conditions of conventional solid–liquid extraction reported in the literature.

Raw Material	Solvent	S:L Ratio (g/mL)	T (°C)	t (min)	Yield	Reference
Freeze-dried and milled saffron flowers	Water:HCl 100:1 (*v*/*v*)	1/30	25	60	13.57 ± 1.16 mg_GAE_/g_DrySF_	[17]
Saffron flowers	Ethanol:Water 59% (*v*/*v*)	1/3	66	104	11.34 ± 3.00 mg_GAE_/g_DrySF_	[30]
Dried and milled saffron flowers	Ethanol:acidified water 50% (*v*/*v*)	1/15	25	1440	1712 mg_CGE_/L	[31]
Dried and milled saffron flowers	Ethanol:Water 25% (*v*/*v*)	1/20	25	1440	1609 mg_CGE_/L	[32]

**Table 3:** Saffron flowers proximate composition.

Moisture (g_H2O_/g_Dry SF_)	Fat (mg/g_Dry SF_)	Ash (mg/g_Dry SF_)	Protein (mg/g_Dry SF_)	Extractives (mg/g_Dry SF_)
5.096 ± 0.001	17.69 ± 0.70	36.28 ± 3.46	45.66 ± 0.30	726.76 ± 10.18

**Table 4:** Eluent gradient.

Time (min)	Eluent A ^1^ (%)	Eluent B ^2^ (%)	Eluent C ^3^ (%)
0	100	0	0
2	100	0	0
5	92	8	0
17	0	14	86
22	0	18	82
29.5	0	21	79
55	0	33	67
70	0	50	50
75	0	50	50
78	20	80	0
81	20	80	0
86	100	0	0

^1^ NH_4_H_2_PO_4_ 50 mM, pH = 2.6; ^2^ 80% acetonitrile + 20% eluent A; ^3^ H_3_PO_4_ 200 mM, pH = 1.5.

**Table 5:** Experimental pretreatment operating variables and extraction kinetics results.

Run	Pretreatment Conditions			Total Polyphenols		Anthocyanins Extraction	
	S:L (g/mL)	T_MW_ (°C)	Energy (kJ/mL)	C_∞_ (mg_GAE_/g_Dry SF_)	u_0_ (mg_GAE_/g_Dry SF_/min)	C_∞_ (mg_GCE_/g_Dry SF_)	u_0_ (mg_GCE_/g_Dry SF_/min)
1	0.50 (0)	80 (0)	0.39	26.62	2.85	6.97	0.58
2	0.50 (0)	80 (0)	0.40	29.00	2.56	8.00	0.58
3	0.50 (0)	100 (+1)	0.51	24.99	2.45	6.97	0.58
4	0.50 (0)	80 (0)	0.37	27.68	3.00	8.43	0.72
5	0.70 (+1)	60 (−1)	0.31	21.05	1.96	5.00	0.56
6	0.30 (−1)	80 (0)	0.35	28.12	10.45	7.94	2.51
7	0.50 (0)	80 (0)	0.36	26.62	1.77	6.70	0.49
8	0.30 (−1)	100 (+1)	0.51	29.53	9.25	8.05	2.38
9	0.70 (+1)	100 (+1)	0.51	24.17	2.57	7.17	0.68
10	0.50 (0)	60 (−1)	0.29	23.01	3.52	9.34	0.97
11	0.30 (−1)	60 (−1)	0.23	23.82	1.62	7.62	0.42
12	0.70 (+1)	80 (0)	0.36	27.64	1.14	6.93	0.34
13	0.70 (+1)	80 (0)	0.40	26.01	7.11	6.52	1.52
14	0.30 (−1)	120 (+2)	0.58	28.62	10.98	5.51	1.83
15	0.70 (+1)	120 (+2)	0.58	25.64	1.03	4.55	0.15
16	0.50 (0)	120 (+2)	0.64	26.68	0.98	5.06	0.18
Control	--	--		26.05	1.23	6.80	0.20

**Table 6:** Extraction results: richness, antioxidant activity (AAO) and color measurement.

Run	TPC Richness (mg_GAE_/g_Dry Extract_)	AC Richness (mg_CGE_/g_Dry Extract_)	DelphinidinRichness (mg/g_Dry extract_)	AAO (mmol_TE_/g_Dry Extract_)	L*	a*	b*	ΔE
1	45.51	11.71	7.43	1.09 ± 0.06	87.54	29.18	−11.23	3.82
2	43.69	11.76	7.71	1.96 ± 0.22	86.97	29.04	−9.46	4.35
3	44.40	9.88	6.04	2.30 ± 0.08	88.49	24.77	−8.78	8.75
4	47.50	13.36	7.58	1.42 ± 0.08	85.78	31.08	−11.53	1.47
5	41.52	10.58	5.99	2.04 ± 0.23	88.80	24.15	−8.83	9.38
6	42.08	11.74	6.37	1.61 ± 0.15	82.07	37.81	−13.43	6.67
7	41.95	10.23	5.49	1.56 ± 0.11	87.96	25.14	−8.98	8.20
8	45.94	12.52	7.34	1.43 ± 0.10	86.44	29.13	−10.78	3.61
9	44.02	12.37	7.89	1.11 ± 0.08	86.98	29.61	−10.72	3.33
10	42.80	14.18	6.12	1.32 ± 0.08	82.79	39.24	−14.82	7.95
11	44.78	12.01	5.03	0.74 ± 0.04	84.82	34.06	−12.58	1.98
12	42.27	10.89	6.28	1.04 ± 0.10	88.94	22.46	−4.46	12.85
13	45.18	11.51	5.38	1.32 ± 0.08	87.89	28.04	−11.36	4.97
14	52.21	10.06	6.64	1.70 ± 0.11	88.63	26.67	−10.43	6.64
15	45.35	8.28	6.49	0.77 ± 0.09	87.81	28.31	−11.34	4.69
16	53.34	9.88	5.98	1.27 ± 0.07	88.41	26.75	−9.94	6.61
Control	41.82	12.00	6.51	1.04 ± 0.04	85.80	32.53	−11.80	0.00

**Table 7:** Optimization results.

Variable	Objective	Range	Relative Importance	Theoretical Optimum	Experimental Optimum
S:L ratio (g/mL)		0.30–0.70		0.30	0.30 ± 0.00
Pretreatment temperature T_MW_ (°C)		60–120		65	66 ± 0
Absorbed Energy Density (kJ/mL)		0.16–0.54		0.24	0.24 ± 0.01
Polyphenol yield (mg_GAE_/g_Dry SF_)	maximize	21.05–29.53	3	29.52	31.33 ± 3.50
Polyphenol initial extraction rate (mg_GAE_/g_Dry SF_/min)	maximize	0.98–10.98	4	10.98	15.24 ± 6.70
Anthocyanin yield (mg_CGE_/g_Dry SF_)	maximize	4.55–9.34	3	6.92	10.19 ± 1.26
Anthocyanin initial extraction rate (mg_CGE_/g_Dry SF_/min)	maximize	0.15–2.51	4	2.38	3.25 ± 1.43
Polyphenol richness (mg_GAE_/g_Dry Extract_)	maximize	41.52–53.34	5	47.18	54.25 ± 3.06
Anthocyanin richness (mg_CGE_/g_Dry Extract_)	maximize	8.28–14.18	5	11.14	12.32 ± 0.87
Antioxidant activity (mmol_TE_/g_Dry Extract_)	maximize	1.26–2.53	3	1.96	2.40 ± 0.16
L*	minimize	82.07–88.94	5	87.38	90.56 ± 0.93
a*	maximize	22.46–39.24	5	29.63	27.60 ± 1.49
b*	minimize	−14.82-(−4.46)	5	−11.18	−16.22 ± 0.45
ΔE	none	1.47–12.85	0	-	8.15

## Appendix A

Wavelengths for color determination:

	**Wavelength (nm)**						
**#**	**X**	**Y**	**Z**	**#**	**X**	**Y**	**Z**
1	424.4	465.9	414.1	16	585	558.5	454
2	435.5	489.5	422.2	17	588.7	561.9	455.9
3	443.9	500.4	426.3	18	592.4	565.3	457.9
4	452.1	508.7	429.4	19	596	568.9	459.9
5	431.2	515.2	432	20	599.6	572.5	462
6	474	520.6	434.3	21	603.3	576.4	646.1
7	531.2	525.4	436.5	22	607	580.4	466.3
8	544.3	529.8	438.6	23	610.9	584.8	468.7
9	552.4	533.9	440.6	24	615	589.6	471.4
10	558.7	537.7	442.5	25	619.4	594.8	474.3
11	564.1	541.4	444.4	26	624.2	600.8	477.7
12	568.9	544.9	446.3	27	629.8	607.7	481.8
13	573.2	548.4	448.2	28	636.6	616.1	487.2
14	577.4	551.8	450.1	29	645.9	627.3	495.2
15	581.3	555.1	452.1	30	663	647.4	511.2
